# Transcatheter device closure of ruptured sinus of Valsalva: Immediate results and short term follow up

**DOI:** 10.4103/0974-2069.52817

**Published:** 2009

**Authors:** Supratim Sen, Amitabha Chattopadhyay, Mily Ray, Biswajit Bandyopadhyay

**Affiliations:** Department of Pediatric Cardiology, Rabindranath Tagore International Institute of Cardiac Sciences, Kolkata, India

**Keywords:** Device closure, ruptured aneurysm, sinus of Valsalva, transcatheter intervention

## Abstract

This is a retrospective, observational study comprising of eight patients with isolated rupture of the sinus of Valsalva (RSOV) who underwent transcatheter device closure. The mean age of presentation was 32.8 ± 10.0 years. New York Heart Association (NYHA) class at the time of presentation was II (six patients) and III (two patients). The RSOVs were all closed using a patent ductus arteriosus device. The mean procedural time was 42.3 ± 5.4 minutes, while the fluoroscopic time was 24.5 ± 6.9 minutes. All had complete closure of the shunt. The average hospital stay was 2.9 ± 1.1 days. There were no major complications. The patients were followed up for a mean of 11.3 ± 4.1 months. At the time of the last follow up all the patients were in NYHA class I. We conclude that in the short term, transcatheter closure of isolated RSOV is a viable alternative to surgical repair.

## INTRODUCTION

Rupture of aneurysm of the sinus of Valsalva (RSOV) is an uncommon condition with a wide spectrum of presentation, ranging from an asymptomatic murmur to cardiogenic shock or even sudden cardiac death.[1] Although the first report of RSOV was in 1839,[2] it was not until 1957 that Lillehei[3] reported the first successful surgical repair. Surgical correction has since become the treatment of choice. However, lately isolated RSOVs have been successfully closed percutaneously using transcatheter devices.[4] At our institution, isolated RSOVs are being treated with the transcatheter technique since 2007.

## METHODS

A retrospective review of medical records from January 2004 to July 2008 revealed that 21 patients were admitted to our institute with RSOV. Of these, eight with isolated RSOV underwent device closure using a patent ductus arteriosus (PDA) occluder (Heartr™ PDA Occluder - Shenzhen Lifetech Scientific Inc.). The remaining 13 patients who underwent surgery required RSOV repair and/or closure of a ventricular septal defect (VSD), and/or an aortic valve replacement. We analyzed the patient demographics, clinical status, echocardiographic and cardiac catheterization findings, procedural variables, and the outcome and subsequent follow up of those who underwent transcatheter closure.

All the patients underwent a detailed clinical evaluation and echocardiographic assessment, to determine their hemodynamic status, the origin and exit site of the RSOV, presence of associated lesions such as VSD and aortic regurgitation (AR), and evaluation of biventricular contractility. After assessing the suitability for device closure, informed consent was obtained. The procedure was done under general anesthesia with transesophageal echocardiographic (TEE) and fluoroscopic guidance. All the patients were heparinized (100 IU/kg) after obtaining vascular access (femoral artery and vein).

**Figure 1:**
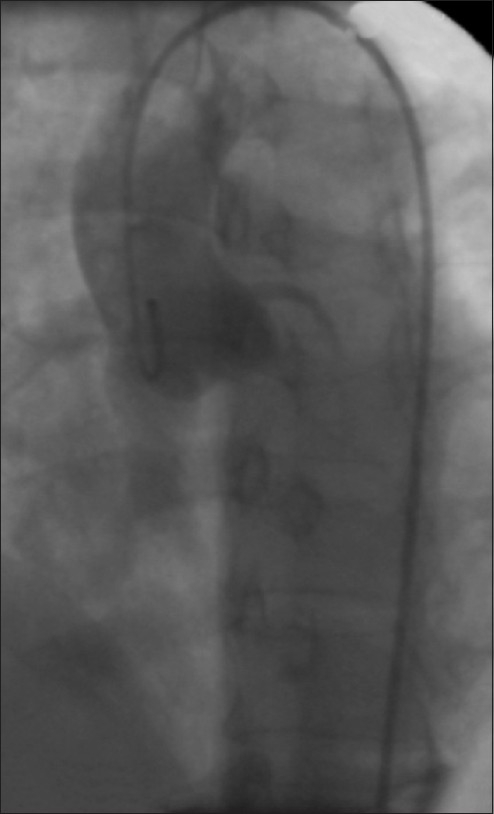
Aortic root injection delineating the ruptured sinus

**Figure 2:**
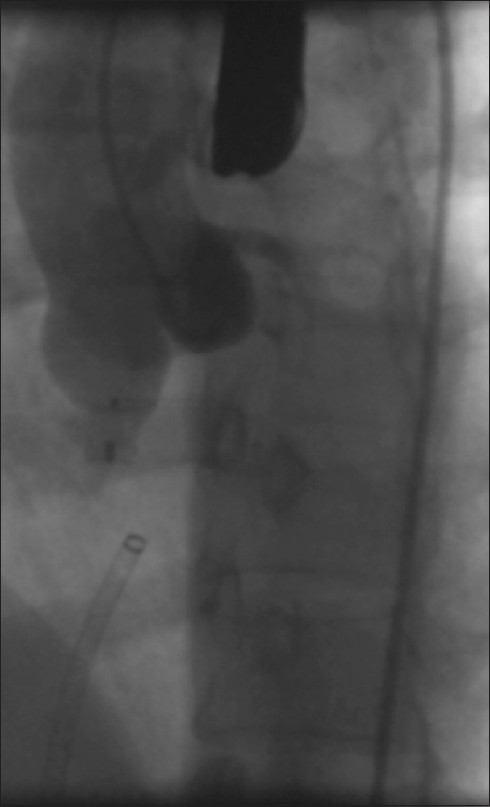
Aortic injection after device placement showing complete closure of the fistulous communication

Detailed TEE evaluation was done prior to proceeding with the hemodynamic study and angiography. This included assessment of the maximum diameter of the aortic end of the RSOV, the minimum diameter and the length of the windsock, and the distance of the aortic end of the RSOV from the coronary ostium. Presence of AR and VSD were also ruled out. An aortogram was done to confirm the TEE findings pertaining to the RSOV and to assess the degree of AR [Figure 1]. A left ventricle angiogram was done to exclude any VSD. The RSOV was crossed with a 6F Judkin Right catheter (Cordis Corporation) and a 0.035” × 260 cm straight tipped Terumo wire (Terumo Corp, Japan) from the aortic side. The wire was manipulated into the superior vena cava (SVC) and snared through the right femoral vein with a 10 mm Goose Neck Snare (Microvena, MN, USA), to form an arteriovenous loop. The delivery sheath was passed from the venous end and pushed over the wire across the RSOV. The device was loaded into the sheath. The aortic retention disc was opened into the ascending aorta and the entire system was pulled back till it anchored at the aortic end of the RSOV. At this point, the other end of the device was delivered by stabilizing the loading cable and pulling back the sheath. The entire maneuver was performed under fluoroscopic and transesophageal echo guidance. A check angiogram was done to confirm the position of the device. Once it was found to be optimum, the device was released. All the patients underwent an aortogram ten minutes post procedure to look for residual flow and to quantify AR [Figure 2]. All the patients were given Aspirin (5 mg/kg/day) for six months following the procedure.

## RESULTS

Eight patients (five males and three females) with RSOV underwent transcatheter device closure during the study period. The mean age was 32.8 ± 10.0 years. All the patients had shortness of breath (NYHA class II - six, III - two) at the time of presentation. The other common symptom was chest pain which was seen in three patients. Two patients (25.0%) presented with acute exacerbation of symptoms and cardiac failure.

The sites of origin and exit of RSOV are summarized in Table 1. The diagnostic transthoracic echocardiography findings correlated completely with intraprocedural procedural TEE and angiographic observations in seven cases (87.5% accuracy).

**Table 1:** Site of origin and exit of rupture of aneurysm of the sinus of Valsalva

Origin	Exit site			
	
	RA	RV	RVOT	Total
Right coronary sinus	2	0	2	4
Non-coronary sinus	3	0	1	4
Left coronary sinus	0	0	0	0
Total	5	0	3	8

RA: Right atrium; RV: Right ventricle; RVOT: Right ventricular outflow tract

The right coronary and noncoronary cusps were sites of origin of the ruptured sinuses, with equal frequency. The site of exit was into the right atrium (five) and right ventricular outflow tract (three).

The PDA devices used for RSOV closure were 10/8 (two), 12/10 (three), and 14/12 mm (three). The mean procedural time was 42.3 ± 5.4 minutes, while the fluoroscopic time was 24.5 ± 6.9 minutes. All patients undergoing device closure were extubated immediately following the procedure. Their average ICU stay was 1.6 ± 0.7 days and the mean length of hospital stay was 2.9 ± 1.1 days. One patient with biventricular dysfunction, acute renal failure, and thrombocytopenia underwent successful RSOV device closure and improved dramatically after the procedure, and was discharged on the fifth day. One patient developed sinus bradycardia with no hemodynamic compromise after device placement, which resolved within a month. Another patient had persistence of mild aortic regurgitation.

There was no procedure-related mortality in our study group and no major complications were noted. Mean duration of follow-up was 11.3 ± 4.1 months. At the time of the most recent follow-up, all the device-closure patients were in NYHA symptom class I. Clinical evaluation revealed stable hemodynamics with absence of murmur in all. Their ECGs did not reveal any ischemia, conduction abnormalities, or rhythm disturbances. Two-dimensional echocardiography revealed the device to be in appropriate position with no evidence of residual shunt, in all the patients. There was no neo-AR. One patient had a mild AR before the procedure, which continued to remain, and was mild at the time of the last follow-up. The biventricular contractility was normal. None of them developed pericardial effusion or thrombus on the device.

## DISCUSSION

Congenital aneurysms of the sinus of Valsalva account for 0.1 to 3.5% of congenital heart defects; the incidence being higher in Asians.[5] They develop as a consequence of incomplete fusion of the distal bulbar septum and truncal ridges, leading to a weakness between the aortic media and the annulus fibrosus of the aortic valve. There is subsequent aneurysmal enlargement at this weak point caused by the high pressure head at the aortic root.[1,5] This dilatation progresses to rupture into the cardiac chambers or mediastinum.[1,4] The aneurysms usually rupture between adolescence and early adulthood.[5] Ruptured aneurysms have been reported more commonly in males, with a male : female ratio ranging from 1.7:1 to 4:1.[2,6] In our series, we had a slight male preponderance (1.7:1).

About 35-58% of patients with RSOV become acutely symptomatic.[7] In our series, only two patients (25.0%) presented with recent worsening of symptoms. The severity of symptoms depends on the rapidity of rupture, the site of rupture, and the size of the defect.[8]

The morbid anatomy of the ruptured sinus in our series differed markedly from the previous studies.[8,9] The right and noncoronary sinuses were the sites of origin with equal frequency, while the commonest site of rupture was the right atrium (62.5%). In comparison, Moustafa *et al*. reported 70 and 25% origin from the right and noncoronary sinuses. In their series, 62% (18 of 29) ruptured into the RV and interventricular septum and 38% into the RA.[8]

The transcatheter technique for RSOV closure was first reported by Cullen *et al*., with a Rashkind umbrella device in 1994. Since then, Gianturco coils, Amplatzer duct occluders, and Amplatzer septal occluders have been used for device closures of RSOV.[4,5]

Our results confirmed a favorable short-term outcome for device closure, with regard to duration of ventilation and hospital stay. All the patients undergoing device closure were extubated immediately post-procedure. Additionally, device closure patients were spared the morbidity related to sternotomy and use of cardiopulmonary bypass. Notably, in our series successful RSOV device closure was accomplished in one patient, where surgery was considered as very high risk, due to biventricular dysfunction, acute renal failure, and thrombocytopenia.

To conclude, transcatheter closure of RSOV is an effective and safe treatment modality for isolated RSOV. In patients where on-pump surgery is high risk, due to poor general condition and comorbidities, transcatheter device closure can be lifesaving. Extended follow-up is required to assess the long-term outcome of these patients.
